# Stability of Middle East Respiratory Syndrome Coronavirus in Milk

**DOI:** 10.3201/eid2007.140500

**Published:** 2014-07

**Authors:** Neeltje van Doremalen, Trenton Bushmaker, William B. Karesh, Vincent J. Munster

**Affiliations:** National Institute of Allergy and Infectious Diseases, National Institutes of Health, Hamilton, Montana, USA (N. van Doremalen, T. Bushmaker, V.J. Munster);; EcoHealth Alliance, New York, New York, USA (W.B. Karesh)

**Keywords:** Middle East respiratory syndrome coronavirus, MERS-CoV, milk, foodborne transmission, stability, viruses, zoonoses, viruses

**To the Editor:** Middle East respiratory syndrome coronavirus (MERS-CoV) was first diagnosed in humans in 2012. Human-to-human transmission of MERS-CoV has been limited, and the transmission route is still unclear. On the basis of epidemiologic studies, involvement of an animal host has been suggested (*1*). Dromedary camels have been identified as a possible intermediate host on the basis of MERS-CoV antibodies and detection of MERS-CoV viral RNA in respiratory swab samples (*1*–*3*). Furthermore, MERS-CoV genome sequences obtained from dromedary camels clustered with MERS-CoV sequences obtained from humans linked to the same farm (*2*). Nonetheless, most persons with MERS-CoV did not report any direct contact with dromedary camels; therefore, how MERS-CoV zoonotic transmission occurs is unclear. MERS-CoV replicates in cell lines originating from a wide variety of different hosts, which suggests the potential for a broader reservoir species range then currently recognized (*4*). However, unlike in dromedary camels, no serologic evidence pointing toward MERS-CoV infection has been found in goats, sheep, and cows (*1*).

Contamination of dairy products has been associated with transmission of bacteria and viruses. Shedding of infectious tick-borne encephalitis virus in milk was detected after experimental infection of goats, and the consumption of raw milk has been associated with tick-borne encephalitis virus clusters (*5*). Similarly, cattle can be infected with foot-and-mouth disease through consumption of raw contaminated milk (*6*).

Here, we investigate the stability of MERS-CoV in dromedary camel milk, goat milk, and cow milk at different temperatures. MERS-CoV strain Jordan-N3/2012 was diluted in unpasteurized milk or nonsupplemented Dulbecco modified Eagle medium (DMEM, GIBCO, Grand Island, NY, USA) to a final median 50% tissue culture infectious dose of 10^5.5^/mL. We placed 1-mL aliquots in screw-cap tubes (Sarstedt, Nümbrecht, Germany) at either 4°C or 22°C and stored them at –80°C at 0, 8, 24, 48, and 72 hours post dilution (hpd) in quintuplicate. Infectious virus titers were determined by endpoint titration on Vero E6 cells in triplicate (*7*). When MERS-CoV was stored at 4°C, the geometric mean of infectious virus titers decreased over 72 hours; we found they decreased 37% (95% CI 0%–62%) in dromedary camel milk, 64% (95% CI 26%–82%) in goat milk, 56% (95% CI 0%–92%) in cow milk, and 80% (95% CI 70%–86%) in DMEM. At 0–72 hpd, virus titers decreased significantly only in goat milk (p = 0.0139, 1-tailed paired *t* test) and DMEM (p = 0.0311) but not in dromedary camel milk (p = 0.1414) or cow milk (p = 0.2895). Samples stored at 22°C showed a greater loss of infectivity than did samples stored at 4°C. Infectious virus titers decreased to <15% when samples were stored at 22°C for 48 hours (loss of 88% [95% CI 67%–96%] for dromedary camel milk, 99% [95% CI 98.6%–99.8%] for goat milk, 98% [95% CI 95%–99%] for cow milk, and 97% [95% CI 87%–99%] for DMEM). This decrease was significant by student 1-tailed paired *t* test analysis comparing *t* = 0 and *t* = 48 hpd (p<0.05). However, despite the reduction in virus titer, viable virus could still be recovered after 48 hours. Pasteurization of raw milk can prevent foodborne disease outbreaks caused by a variety of pathogens. We heat-treated dromedary camel, cow, goat milk, and DMEM samples for 30 min at 63°C, after which no infectious virus could be recovered (Figure).

**Figure:**
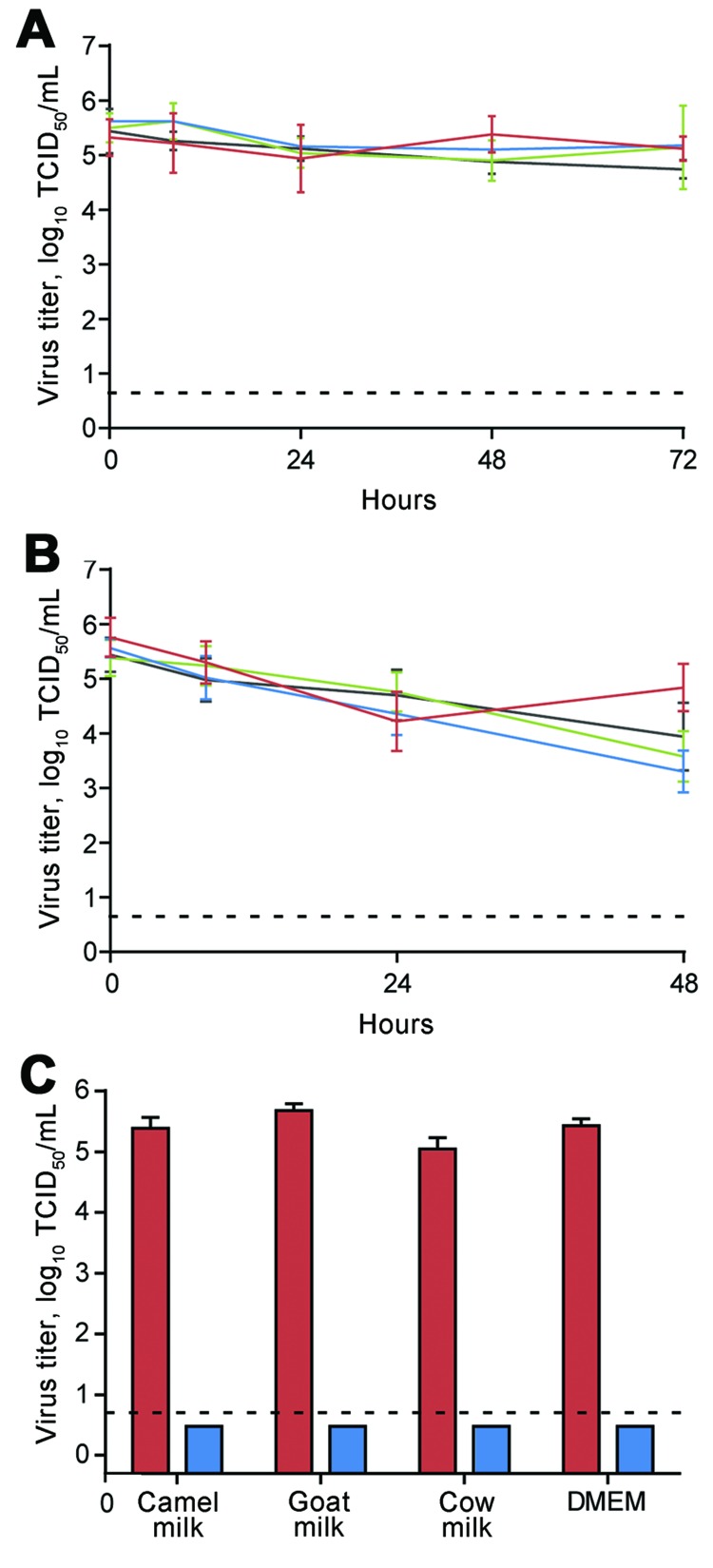
Viability of MERS-CoV in unpasteurized milk. MERS-CoV strain Jordan-N3/2012 was diluted in milk or DMEM to a final TCID_50_ of 10^5.5^/mL and stored at either 4°C (A) or 22°C (B). MERS-CoV titer was determined at 0, 8, 24, 48, and 72 hours post dilution in quintuplicate. Red indicates dromedary camel milk; blue indicates goat milk; green indicates cow milk; black indicates DMEM. C) Milk containing MERS-CoV was pasteurized by heating 1-mL aliquots of diluted virus at 63°C for 30 min in triplicate. Red indicates unpasteurized; blue indicates pasteurized. Infectious virus titers were determined by endpoint titration on Vero E6 cells in triplicate. Dotted line depicts the detection limit of the assay. MERS-CoV, Middle East respiratory syndrome coronavirus; TCID_50_, 50% tissue culture infective dose; DMEM, Dulbecco modified Eagle medium.

CoV survival has been studied in phosphate-buffered saline and minimal essential media and, like MERS-CoV, human coronaviruses–229E and -OC43 and severe acute respiratory syndrome–CoV were able to survive in suspension at room temperature for several days (*8*,*9*). Moreover, severe acute respiratory syndrome–CoV was completely inactivated after heat treatment at 60°C for 30 min (*9*).

Human-to-human transmission of MERS-CoV is inefficient, and the transmission route has not yet been revealed. The predominant detection of MERS-CoV by quantitative PCR in nasal swab samples suggests the virus causes upper respiratory tract infection in dromedary camels (*3*). Which route or combination of routes is responsible for its zoonotic transmission is unclear, and foodborne transmission should not be excluded. Residents of the Arabian Peninsula commonly drink unpasteurized milk. Our results show that MERS-CoV, when introduced into milk, can survive for prolonged periods. Further study is needed to determine whether MERS-CoV is excreted into the milk of infected dromedary camels and, if so, whether handling or consuming contaminated milk is associated with MERS-CoV infection. Recently Nipah virus was transmitted experimentally by drinking , which resulted in respiratory tract rather than intestinal tract infection (*10*). A similar transmission mechanism for MERS-CoV could result in contamination of the oral cavity and subsequent infection of the lower respiratory tract. Pasteurization of milk can prevent foodborne transmission (*9*). We showed that heat treatment decreased infectious MERS-CoV below the detection limit of our titration assay, and this might function as a relatively easy and cost-effective measure to prevent transmission.
